# Bioprinting of Microtissues Within Mechanically Tunable Support Baths to Engineer Anisotropic Musculoskeletal Tissues

**DOI:** 10.1002/advs.202509313

**Published:** 2026-02-06

**Authors:** Francesca D. Spagnuolo, Gabriela S. Kronemberger, Daniel J. Kelly

**Affiliations:** ^1^ Trinity Centre for Biomedical Engineering Trinity Biomedical Sciences Institute Trinity College Dublin Dublin Ireland; ^2^ Department of Mechanical Manufacturing and Biomedical Engineering School of Engineering Trinity College Dublin Dublin Ireland; ^3^ Department of Anatomy and Regenerative Medicine Royal College of Surgeons in Ireland Dublin Ireland; ^4^ Advanced Materials and Bioengineering Research Centre (AMBER) Royal College of Surgeons in Ireland and Trinity College Dublin Dublin Ireland

**Keywords:** anisotropy, bioprinting, microtissues, stiffness, support bath

## Abstract

Bioprinting is a powerful tool for engineering living grafts, however replicating the composition, structure and function of native tissues remains a major challenge. During morphogenesis, cellular self‐organization and matrix development are strongly influenced by the mechanical constraints provided by surrounding tissues, suggesting that such biophysical cues should be integrated into bioprinting strategies to engineer more biomimetic grafts. Here, we introduce a novel bioprinting platform that spatially patterns mesenchymal stem/stromal cell (MSC)‐derived microtissues into mechanically tunable support baths. By modulating the bath's mechanical properties, we can precisely control the physical constraints applied post‐printing, directing both filament geometry and cellular behavior. Support bath stiffness regulated mechano‐sensitive gene expression and microtissue phenotype, with softer matrices favoring chondrogenesis and stiffer environments promoting (myo)fibrogenic differentiation. In addition, the physical properties of the non‐degradable support bath modulated microtissue fusion and extracellular matrix organization, with increased collagen fiber alignment in stiffer baths. Leveraging these findings, it was possible to engineer either articular cartilage, meniscus, or ligament grafts with user‐defined collagen architectures by simply varying the physical properties of the support bath. This platform establishes a foundation for bioprinting structurally anisotropic and phenotypically distinct constructs, thereby enabling the scalable engineering of a range of different musculoskeletal tissues.

## Graphical abstract

This study presents a novel 4D bioprinting platform for engineering biomimetic musculoskeletal grafts. By tuning the mechanical properties of support baths, we enhance tissue fusion, collagen alignment, and cell differentiation. Using this strategy, we successfully fabricate scaled‐up, anisotropic tissues such as meniscus, articular cartilage, and ligament. This platform offers new solutions for advanced regenerative medicine applications.

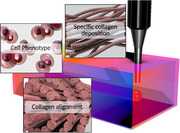

## 1 Introduction

The field of tissue engineering (TE) seeks to regenerate tissues or organs impaired by trauma or disease. Achieving this goal will require the engineering of regenerative grafts that replicate the complex structure, composition, and function of the native tissue. This is particularly true for the repair of musculoskeletal tissues, such as articular cartilage (AC), meniscus, ligament, and tendon, whose unique biomechanical function derives from the specialized architecture of their extracellular matrix (ECM). Damage to these tissues commonly initiates further joint degeneration, leading to the development of debilitating diseases such as osteoarthritis (OA) [1, 2]. Previous attempts at engineering such tissues have focused on seeding stem/progenitor cells within a 3D scaffold or a hydrogel, which are then stimulated in an attempt to promote tissue‐specific ECM deposition [3, 4, 5, 6]. However, these strategies typically fail to produce phenotypically defined tissues with biomimetic collagen network architectures that are integral to their physiological function, dramatically limiting their clinical utility.

Limitations associated with traditional TE strategies have motivated increased interest in scaffold‐free approaches for the development of regenerative grafts. These strategies harness the ability of cells to self‐organize in response to cell–cell and cell‐matrix interactions, mimicking the self‐organization observed in polarized tissues during embryonic development and during adult tissue self‐renewal [7]. Under specific physicochemical conditions, key aspects of such self‐organization can be replicated in vitro, allowing stem cells to generate microtissues (µTs) or organoids that mimic key features of a specific tissue or organ [8]. Under the appropriate conditions, it may be possible to combine such biological building blocks to engineer scaled‐up tissue grafts [9, 10, 11, 12]. Realizing this goal will require the development of biofabrication strategies that can not only control the phenotype of the resulting graft but can also direct its (re)modelling to enable the engineering of tissues with user‐defined ECM architectures.

During development, individual tissues almost never develop in isolation, but do so concurrently with surrounding tissues and organs, which mechanically confine, impinge upon, or pull on them [13, 14]. These mechanical influences have been postulated to affect the development of different tissues and indeed the entire early mouse embryo [15]. When cultured in vitro in the presence of physical boundaries, cells maintain the ability to sense and respond to such geometrical and physical cues [16]. For example, endothelial cell sheets exhibit geometry‐dependent growth behavior: on patterned substrates (i.e., squares or annular rings), proliferation is suppressed in the densely packed regions but persists at the periphery and in the mechanically stressed areas such as edges and corners. Similarly, the differentiation of adult mesenchymal stem/stromal cells (MSCs) is strongly influenced by geometric confinement and substrate stiffness [17, 18, 19, 20], with stiffer substrates and increased cytoskeletal tension promoting an osteoblastic phenotype [21]. These geometric and mechanical cues provide an additional layer of signaling, conveying positional information to distinct cells and guiding the maturation and organization of tissues and organs [22]. Therefore, by providing spatially physical boundary conditions to populations of µTs or organoids, it may be possible to direct their fusion and (re)modelling in a programmable manner. If this can be achieved at scale, it would facilitate the transition of multiple individual µTs into scaled‐up constructs that recapitulate the anatomical shape, internal structure, composition, and function of specific tissues and organs.

3D bioprinting is now an established technique to spatially pattern cells and supporting biomaterials to replicate key anatomical features of musculoskeletal tissues [23, 24] and engineer anisotropic musculoskeletal tissues. Such approaches have recently been extended to the bioprinting of cellular aggregates, µTs, and organoids [25, 26, 27, 28]. However, current bioprinting approaches often struggle to achieve precise microtissue patterning and adequate fusion between µTs [29], which is critical requirement for the engineering of scalable grafts. Moreover, little consideration has been given to how the bioprinting process can be used to direct long‐term phenotype and structural (re)modelling of the resulting tissues. This could potentially be addressed using emerging bioprinting concepts, such as the use of sacrificial or temporally adapting inks, but particularly the use of support baths that provide precise biophysical cues to printed cells [30, 31, 32, 33, 34, 35]. Previous studies have utilized such support baths to enable the high resolution (bio)printing of low‐viscosity (bio)inks [36], whereby print quality is determined by the physical properties (e.g. shear thinning, yield stress, stiffness) of the bath [37, 38]. For instance, the freeform reversible embedding of suspended hydrogels (FRESH) method employs extrusion into a gelatin slurry to support high resolution 3D printing of a range of different inks [39, 40, 41], however such support baths are typically designed to be removed post‐printing and are not suitable for providing well defined physical constraints to bioprinted cells. This can potentially be addressed using baths that can be chemically crosslinked post‐printing, thereby physically supporting the resulting construct during in vitro culture [42, 43, 44]. Xanthan gum (XG) has previously been used as a support bath for bioprinting [45] and can be further functionalized using methacrylate to create a shear‐thinning and self‐healing photocrosslinkable bath [46]. The highly tunable nature of XG suggests that this material could be used as a support bath to not only facilitate the extrusion bioprinting of µTs or organoids, but to also modulate their physical environment post‐printing.

Recognizing that normal tissue development depends on both the self‐organizing potential of stem cells and key physicochemical cues from the microenvironment to establish its final architecture and function, here, mechanically tunable methacrylated xanthan gum (XG‐MA) support baths will be used to control the surrounding matrix stiffness and the degree of physical confinement experienced by MSC‐derived µTs post‐printing. To this end, an extrusion‐based bioprinting platform will be used to precisely pattern µTs at a high density within a XG‐MA support bath. By precisely tuning the physical properties (rheology, stiffness) of the support bath, we demonstrate that it is not only possible to modulate print fidelity, but to also direct microtissue fusion, differentiation, and the long‐term structural organization of the bioprinted graft.

## 2 Results and Discussion

### 2.1 Development of a Temporally Adapting Support Bath for Extrusion Bioprinting of Microtissues

To facilitate the bioprinting of large numbers of µTs into a support bath, we first needed to identify a sacrificial supporting bioink that would rapidly vacate the support bath to enable microtissue fusion post‐printing. Gelatin has previously been used as a sacrificial ink in support baths, enabling the 3D printing of perfusable channels for vascular TE [47, 48]. Therefore, we first assessed the rheological properties of gelatin at a range of different concentrations (Figure S1A). While all gelatin concentrations displayed favorable shear thinning properties, we selected a relative low concentration (1% w/v) to minimize the shear stresses experienced by the µTs during the bioprinting process. Another key advantage of gelatin is its temperature‐dependent viscosity, which displays a higher viscosity at lower temperatures. This property prevents the settling of µTs within the print cartridge when cooled to 4°C for 15 min before printing. To determine whether the gelatin would vacate the printed channel by diffusing into the support bath, we performed a perfusion test using black ink after printing the gelatin ink (Figure S2). Within 15 s, the ink was able to flow through the void channel left by the liquefied gelatin, confirming its sacrificial behavior when printed into the support bath.

**FIGURE 1:**
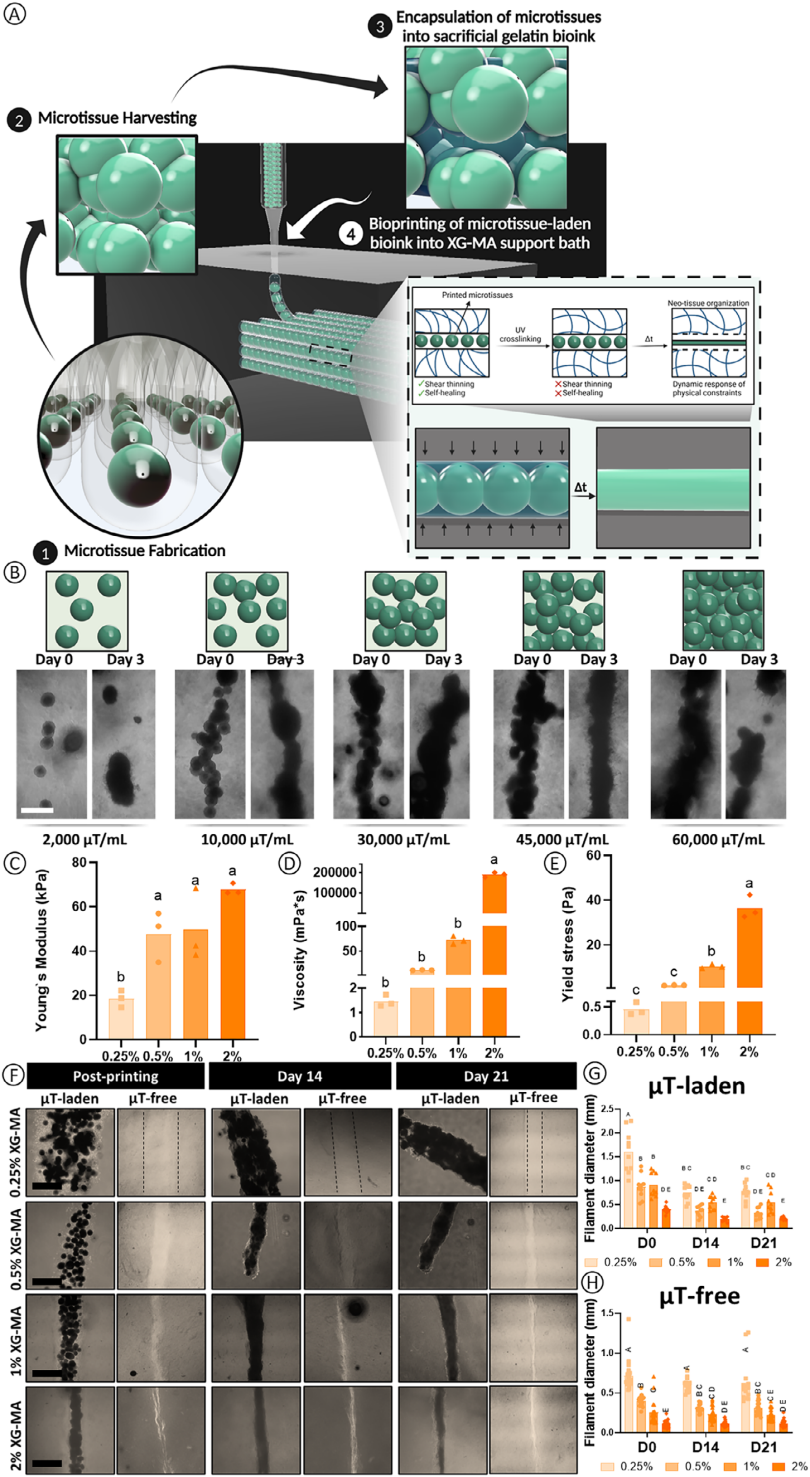
The concentration of the support bath influences the extent of filament confinement over time post‐printing. A) Graphical abstract of the bioprinting platform. µTs are fabricated using a high‐throughput agarose microwell system and cultured for 2 days prior to their harvest and encapsulation into a gelatin bioink and extrusion into an XG‐MA support bath. After UV‐crosslinking, the filament diameter changes over time due to the changing physical confinement provided by the support bath. B) The influence of µTs density on the development of a continuous tissue filament 3 days post‐printing. Scale bar (Sb): 200 µm. C) Young`s Modulus of XG‐MA hydrogels following UV crosslinking for 4 min. D) Viscosity of the un‐crosslinked XG‐MA bath at different concentrations. E) Yield stress of the un‐crosslinked XG‐MA bath. F) Brightfield microscope imaging of the printed µTs within XG‐MA support baths of differing concentrations at different time points. Scale bar: 1 mm. Filament diameter changes over time for the (G) µT‐laden inks and the (H) µT‐free inks. For graphs C), D), and E) a one‐way ANOVA followed by Tukey`s multiple comparisons tests was performed to assess the differences among groups. For graphs G) and H), a 2‐way ANOVA followed by the Tukey`s post hoc comparison test was performed to assess the differences between the groups at different time points. For all graphs included in this figure, significance was accepted when *p*<0.05. Statistically comparable groups are labeled using compact letter display where groups sharing the same letter are not significantly different (*p*>0.05).

In order to generate a continuous filament of tissue, we next needed to determine how the distribution of µTs post‐bioprinting was influenced by their density within the supporting gelatin bioink. The ideal µT density for extrusion bioprinting will depend on the needle size and the µT dimensions, with high densities hindering extrusion [49] or imposing the cells to very high shear stresses, thereby negatively impacting viability [50, 51]. Here, we found that µTs measuring 175 ± 5 µm in diameter (Figure S1C) could be extruded at relatively high densities using a 22G metal needle (outer diameter of 0.7 mm and an inner diameter of 0.41 mm). Bioprinting at low µT densities (2,000 µT mL^−1^) resulted in clear gaps within the resulting filament (Figure 1B), while higher densities (60,000 µT mL^−1^) caused clogging during printing, which also resulted in the development of gaps (Figure 1B). We identified 45,000 µT mL^−1^ as the optimal density to form compact, continuous tissue filaments post‐printing (Figure 1B).

To enable the high‐resolution bioprinting of µTs, we next sought to extrude them into a support bath with tailorable mechanical and rheological properties. Previous studies have typically focused on the printability of bioinks and on post‐printing outcomes from a purely geometric perspective. Here we sought to combine a sacrificial bioink (1% gelatin) with a non‐sacrificial support bath (XG‐MA), thereby creating mechanically tunable boundary conditions to direct cell–cell and cell–matrix interactions, ultimately regulating phenotype and structural organization of resulting tissue. To generate a non‐sacrificial support bath, it was essential to utilize a material that could be crosslinked post‐printing, thereby stabilizing the printed structure throughout the in vitro culture. This is in contrast with other commonly used support baths, which are designed to provide only temporary physical support during the printing process and to rapidly degrade post‐printing [39, 40, 41, 45]. We selected xanthan gum (XG) as a support bath and further modified it with a methacrylate group (XG‐MA) to stabilize and mechanically support the printed filament throughout the culture process (Figure S1A). The reaction between glycidyl methacrylate and the hydroxyl or carboxyl groups of XG was confirmed through ^1^H‐NMR analysis, where the presence of the two peaks at 6.58 and 6.17 ppm (Figure S1A) confirms the modification of the XG chain with a methacrylate (─C═C) group, enabling the material to be crosslinked post UV exposure. 1.5% XG or 1% XG‐MA have previously been used as a support bath for bioprinting [45, 46]. To expand on this, we tested a range of XG‐MA concentrations (from 0.25% to 2% w/v) and measured their stiffness post UV crosslinking. We found that the Young's modulus increased with XG‐MA concentration, from 20 kPa for the softest bath to 65 kPa for the stiffest bath (**p < 0.01; Figure 1C).

We also investigated the shear thinning and thixotropic properties of the support baths to assess their suitability for bioprinting applications (Figure S3). All tested concentrations of XG‐MA displayed shear‐thinning (Figure S3A,B) and thixotropic (Figure S3C) properties, allowing the bath to maintain its structural integrity post‐printing. Irrespective of concentration, the XG‐MA was able to recover after experiencing high shear stress, with a faster recovery observed in the higher concentration baths (Figure S3C). Having developed a support bath with tunable mechanical and rheological properties, we next sought to assess its capacity to support high resolution extrusion bioprinting of high density microtissue‐laden bioinks. We first sought to assess how the concentration of the support bath influenced the geometry of the resultant print following extrusion bioprinting of a microtissue‐laden gelatin ink and a microtissue‐free gelatin ink. Using the same extrusion printing parameters (6 uL s^−1^ extrusion rate, 2 mm s^−1^ printing speed), a higher concentration XG‐MA support bath enabled a higher printing fidelity/resolution (i.e., a smaller printed filament diameter) for both a µT‐free and a µT‐laden ink (Figure 1G,H). The printed µTs generated a larger diameter filament when printed into a softer bath, ranging from 1.5 to 2 mm in diameter following extrusion into a low stiffness 0.25% bath, to 0.25–0.5 mm in the stiffer 2% bath for both µT‐laden (Figure 1G) and µT‐free inks (Figure 1H). 7 days post‐printing, the µTs appeared to have fused, with high cell viability observed in all support baths (Figure S5A). This suggests that the shear stress applied to the cells within the µTs during the bioprinting process, as well as UV exposure to crosslink the support bath post‐printing, does not negatively impact cell viability.

Not only did the concentration of XG‐MA in the support bath influence the initial diameter of the printed filament, but it also impacted how it changed with time in culture. In µT‐laden bioinks (µTs + 1% gelatin), the filament diameter was found to reduce in diameter with time in culture, with larger absolute reductions observed in the softer baths (Figure 1F,G). This is likely due to both cellular remodeling and physical forces exerted by the support bath on the printed µTs as the supporting gelatin ink washes out with time (Figure S2). However, even in the µT‐free prints (1% gelatin) ‐where no cellular remodeling is involved‐ a reduction in filament diameter over time is observed, pointing to an active role for the support bath in determining the long‐term geometry of the print (Figure 1F,H). Collectively, these results suggest that the support bath exerts a radially compressive force on the filament post‐printing, resulting in a gradual reduction in diameter until an equilibrium is reached between day 14 and 21 (Figure 1H). To assess the smoothness of printed microtissue filaments, we quantified variations in filament diameter from brightfield microscopy images (Figure S4A). For each filament, five measurements were taken along its length, and the standard deviation of these values was used as an index of smoothness. Lower standard deviations reflect more uniform diameters (smoother filaments), while higher values indicate greater irregularity (Figure S4B). As fusion progressed, this standard deviation decreased, consistent with increased tissue compaction. Notably, even immediately after printing, filaments in the stiffer support baths (1% and 2% XG‐MA) displayed lower standard deviations, indicating higher initial smoothness. This suggests that the physical properties of the bath contribute to filament compaction and smoothness, complementing the filament diameter analysis (Figure S4B).

These results suggest that the XG‐MA support bath can be used to support the bioprinting of µTs, where the printed structures will change in shape, function, and/or behavior over time in response to external stimuli provided by the bath. Different printing approaches show how a more dynamic environment can support the maturation of tissue constructs [52, 53, 54]. For example, programming delayed dissolution into sacrificial bioinks has previously been used to provide precise control over the spatial and temporal introduction of architectural features into cell‐laden hydrogels, which was leveraged to support the development of a pre‐vascularized, bone‐like graft [31]. Other approaches leverage cell‐generated forces within bioinks to control shape‐morphing in support baths post‐printing [55]. In contrast, in our system, the extent of physical confinement generated by the different support baths over time would appear to guide µT fusion, compaction, and alignment of the printed filament. Previous studies have shown that such physical boundaries can guide neo‐tissue organization, enabling the engineering of anisotropic living systems [56, 57]. Furthermore, tissue maturation in vivo is driven by spatiotemporal cellular signaling and mechanical forces [58]. Therefore, we hypothesized that the degree of physical confinement imposed by the bath over time would influence µT fusion and phenotype, as well as the structural organization of the resulting tissue, thereby enabling the bioprinting of anisotropic grafts.

### 2.2 YAP Activation in MSC Derived Microtissues is Influenced by Support Bath Stiffness

**FIGURE 2:**
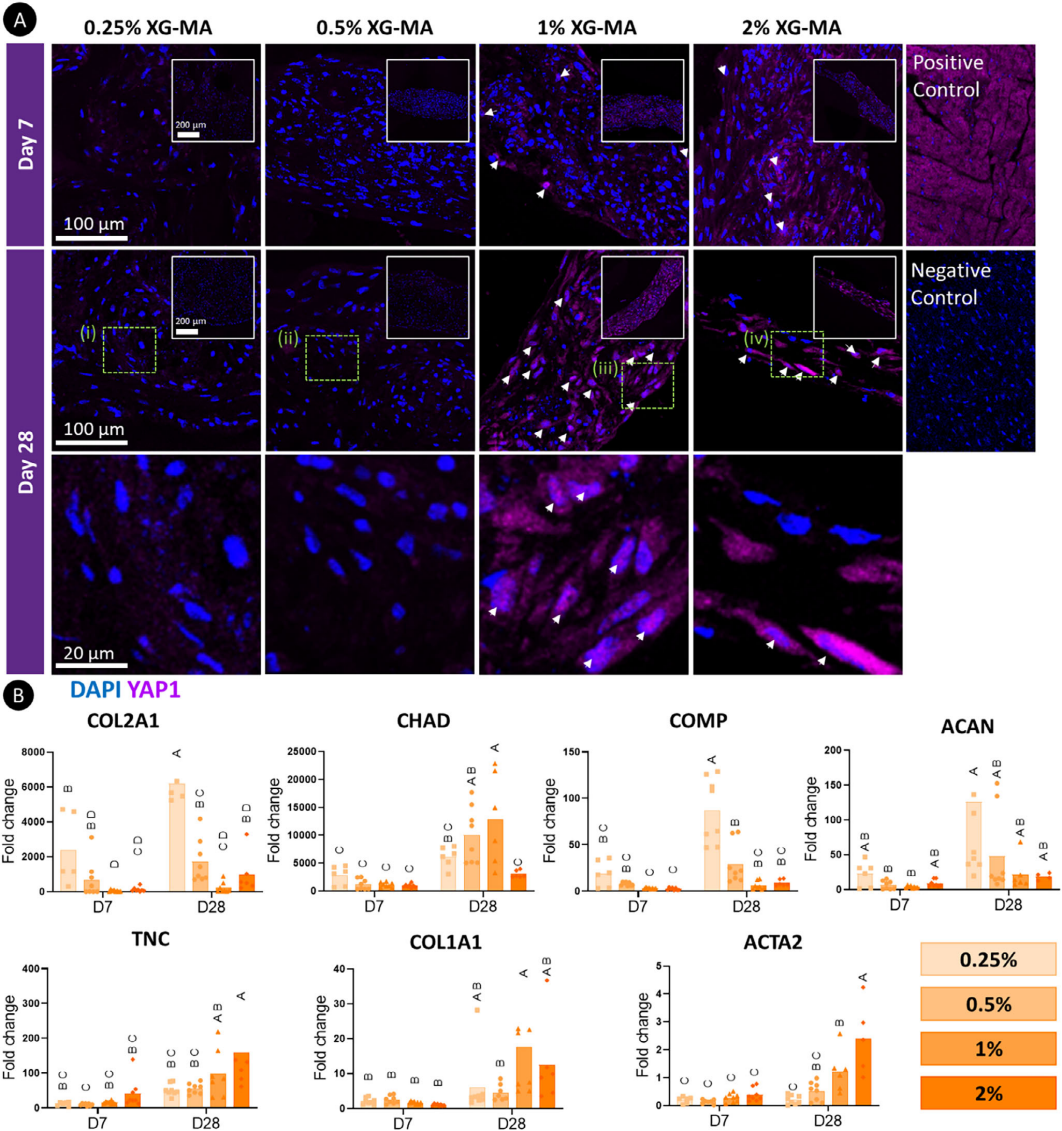
The physical properties of the support bath influence MSCs phenotype. A) YAP1 staining of printed µTs reveals spatial differences in YAP expression depending on the stiffness of the support bath. White box insets show a larger area of the printed filament. Scale bar: 200 µm. White arrows indicate regions where YAP colocalizes with the nuclei, indicating YAP activation. Magnified views are provided for the regions highlighted by the green dashed boxes, see panels i), ii), iii), and iv) for each of the support baths. Positive control is mouse heart and negative control is mouse heart without primary antibody. Scale bars: 100 and 20 µm B) Expression of a range of genes associated with cartilage (COL2A1, CHAD, COMP, ACAN), fibrocartilage or ligament tissue (COL1A1, TNC, ACTA2). For statistical analysis, a two‐way ANOVA test was performed followed by Tukey's post comparison to compare the means of each group at different time points. Significance was accepted when *p*<0.05. Statistically comparable groups are labeled using compact letter display where groups sharing the same letter are not significantly different (*p*>0.05).

MSCs are known to respond to the stiffness of their surrounding environment [59, 60]. As the stiffness of the support bath increased with XG‐MA concentration (Figure 1C), we next investigated whether such differences in the local mechanical environment would influence the activity of known mechanosensitive proteins in the bioprinted µTs (Figure 2A). The activation of YAP, a key protein in the Hippo signaling pathway that regulates growth and development, is known to be influenced by mechanical cues in the ECM for diverse cell types, including cardiac cells, myofibroblasts, and MSCs [61, 62, 63, 64, 65, 66]. YAP is regulated by actomyosin cytoskeletal tension and functions as a transducer of mechanical cues, with increased ECM stiffness and cell spreading leading to higher YAP activity [21]. This, in turn, can influence cellular differentiation, with YAP activation known to promote osteogenesis [67], while suppressing adipogenic [68] and chondrogenic differentiation [65]. Here, bioprinted µTs were maintained in media supplemented with TGF‐β3 post printing, a growth factor known to support either a chondrogenic or a myogenic phenotype in MSCs, depending on other environmental factors such as the ability of cells to spread [58, 68, 69, 70]. To assess YAP activity across different bioprinting conditions, we performed YAP staining at both early and late time points in constructs printed into XG‐MA support baths of differing stiffness (Figure 2B). YAP activity was significantly higher in µTs printed into the stiffer support baths (1% and 2% XG‐MA). In these stiffer conditions, greater YAP nuclear colocalization was also observed (Figure 2A, white arrows). This suggests that the greater stiffness of higher concentration XG‐MA baths promotes YAP transcription and its activation.

### 2.3 Support Bath Stiffness Regulates Microtissue Phenotype

**FIGURE 3:**
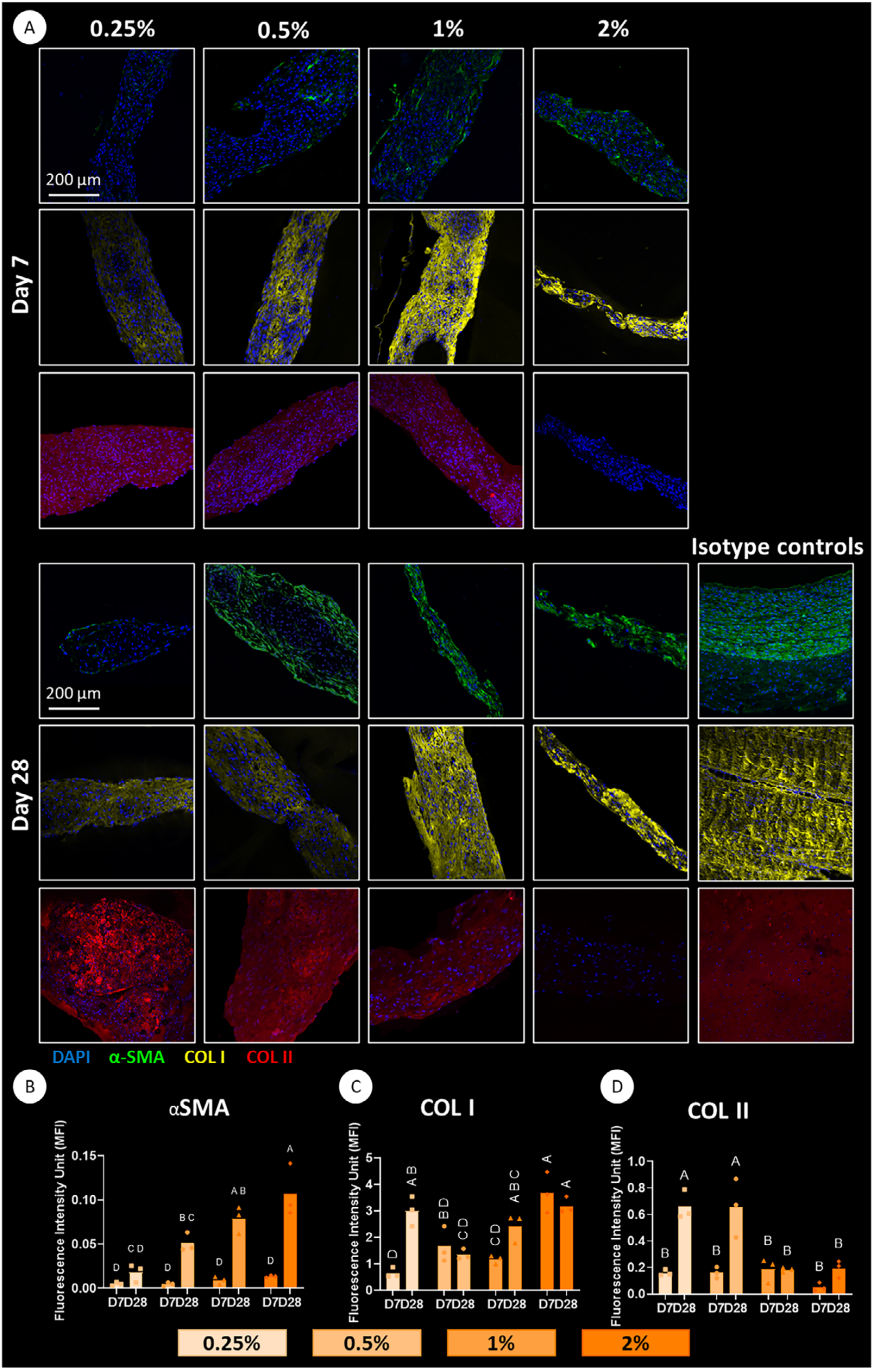
Immunofluorescence staining for specific proteins within the bioprinted microtissues. (A) Staining for type I collagen, type II collagen, and α‐SMA deposition in 7‐ and 28‐day post‐printing in the different support baths. Positive controls are arterial control for α‐SMA, ligament for type I collagen, and articular cartilage for type II collagen. Scale bar: 200 µm. Quantification of immunofluorescence intensity normalized with background intensity and normalized to the number of nuclei of each region of interest (ROI) for αSMA (B), collagen I (C), and collagen II (D); *n* = 3. For all the graphs, a two‐way ANOVA test was performed, followed by Tukey's post hoc comparison to compare the means of each group at different time points. Significance was accepted when *p*<0.05. Statistically comparable groups are labeled using compact letter display where groups sharing the same letter are not significantly different (*p*>0.05).

It is well established that physical and biochemical signals work in synergy to guide cell fate and function [71, 72]. As YAP activity within the bioprinted µTs was strongly regulated by the stiffness of the surrounding support bath, we further hypothesized that longer‐term MSC phenotype would also depend on these physical cues. To this end, the printed constructs were cultured in the presence of TGF‐β3, and MSC phenotype was assessed over a 4‐week culture period using a range of biochemical and histological assays. We first assessed the expression of a range of genes associated with chondrocytes, fibrochondrocytes, and myofibroblasts (Figure 2C). Actins are major cytoskeletal proteins involved in cell migration and contraction. Alpha smooth muscle actin (α‐SMA) is particularly associated with contractile fibroblastic cells such as myofibroblasts, which play a role in tendon and ligament healing and maintaining tissue homeostasis [73]. The highest levels of ACTA2 expression (a gene that encodes for α‐SMA) were observed in the stiffer 2% bath after 28 days of culture (Figure 2C). We also assessed α‐SMA deposition through immunofluorescence staining (Figure 3A). While weak α‐SMA staining was observed at day 7 (Figure 3A), a significant increase was evident by day 28, particularly in the stiffer (1% and 2%) baths (*p*<0.05) (Figure 3A,B). COL1A1 and TNC (Tenascin‐C) gene expression, markers associated with a ligamentous or tendon phenotype, was also higher in the stiffer baths (Figure 2C). In agreement, type I collagen deposition was generally higher in the stiffer support baths at both early and late time points (Figure 3A,C).

While genes and proteins associated with a ligament or tendon phenotype were more highly expressed in the stiffer bath, the expression of the chondrogenic genes COL2A1, COMP, and ACAN was higher in µTs bioprinted into the softer support baths (Figure 2C). In agreement with this finding, strong staining for type II collagen deposition was observed in the tissues generated in the soft baths, with little deposition observed in the stiffer baths (Figure 3A,D). Together, these findings suggest that the MSC‐derived µTs actively respond to the mechanical cues provided by the different support baths. The softer 0.25% and 0.5% XG‐MA support baths promoted greater collagen II deposition and reduced α‐SMA (ACTA2) expression, resulting in a more chondrogenic phenotype (Figure 3). The observation of enhanced sGAG accumulation in these softer environments further confirms this conclusion (Figure S5B,C). The 1% XG‐MA bath, which possesses an intermediate stiffness, appeared to support a more fibrochondrogenic phenotype, as evident by simultaneous sGAG (Figure S5B) and type I collagen deposition (Figure 3A,C). Finally, the stiffest 2% XG‐MA baths supported the highest levels of type I collagen production (Figure 3A,B) but little sGAG deposition (Figure S5B), suggesting the development of a more ligamentous tissue.

### 2.4 Support Bath Stiffness Regulates Collagen Fiber Alignment in Bioprinted Tissues

**FIGURE 4:**
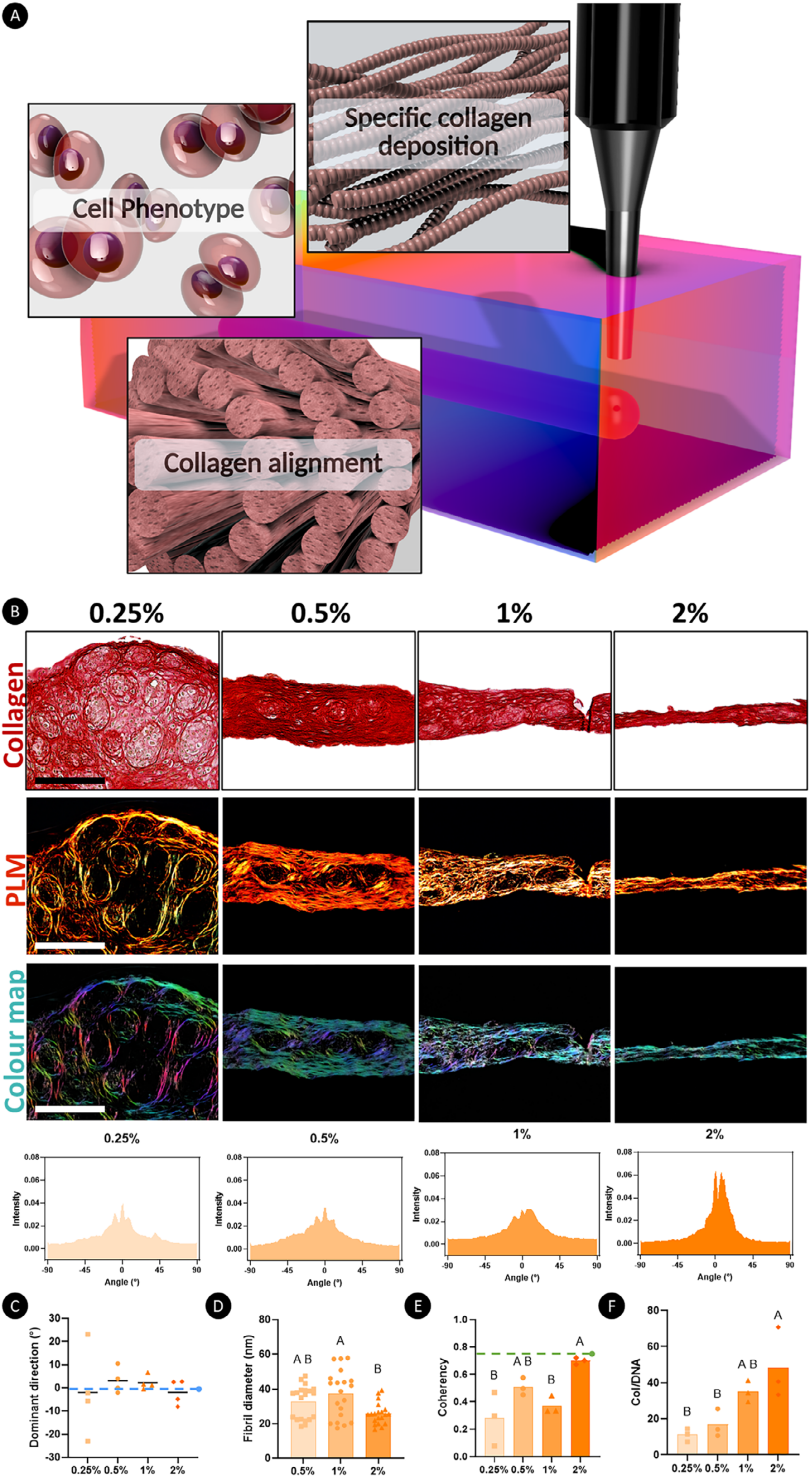
A stiffer support bath improves microtissue fusion and the alignment of the secreted collagen fibers. A) Graphical abstract of a bioprinting platform capable of generating phenotypically distinct musculoskeletal grafts. B) Histological assessment of bioprinted µTs after 28 days post‐printing with picrosirius red staining, using PLM imaging to analyze collagen fiber alignment. Color maps are generated from PLM images. Color hue represents fiber orientation, with blue/cyan corresponding to fibers aligned at 0° and pink/red corresponding to fibers aligned at 90°. Sb: 200 µm. Quantification of the fiber orientation was then performed for the 0.25%, 0.5%, 1% and 2% XG‐MA support baths. C) Average collagen fiber orientation with the filaments generated in the different support baths. D) Fibril diameter at 28 days post‐printing. E) Fiber coherency was quantified, with values approaching 1 indicating highly aligned fibers and values closer to 0 reflecting greater dispersion in multiple directions (*n* = 4). F) Biochemical quantification of total collagen normalized to DNA levels. Statistical difference is determined using a two‐way ANOVA test with Tukey's post hoc comparison test. Significance was accepted with *p*<0.05. Statistically comparable groups are labeled using compact letter display where groups sharing the same letter are not significantly different (p>0.05).

Encouraged by the successful bioprinting of high density µT‐laden bioinks into a temporally adapting support bath, where the µTs fused effectively over time and adopted a phenotype defined by the stiffness of the surrounding support bath, we next sought to explore if this bioprinting platform could be used to direct collagen organization and hence enable the engineering of anisotropic soft tissues. The bioprinting of such structurally organized tissues has previously been demonstrated using collagen‐based bioinks, where the extrusion process directed collagen fiber alignment within the inks, which in turn facilitated cell spreading and alignment along the fiber direction, thereby promoting the formation of anisotropic tissue [74, 75, 76]. However, the slow gelation and relatively poor printability of collagen‐based bioinks challenge this approach [77, 78, 79]. Here, we sought to guide collagen alignment by printing µTs into a physically constrained environment that facilitates µT fusion and directs subsequent ECM organization. To validate our ability to bioprint custom‐shaped, highly aligned musculoskeletal tissues, we employed polarized light microscopy (PLM) to assess the preferential alignment of collagen fibers along the printed filaments (Figure 4). After four weeks of in vitro culture, the µTs fused to generate filaments of tissue whose internal structure was directed by the physical constraints imposed by the XG‐MA support bath. Picrosirius red (PR) staining (Figure 4A) revealed robust collagen deposition across all groups, with more homogeneous collagen distribution observed within filaments printed in the stiffer baths (Figure 4A). Collagen deposition, quantified relative to DNA levels (Figure 4E), was also significantly higher in the stiffer baths (*p*<0.01). In addition, the XG‐MA bath provided effective boundary conditions for directing collagen organization in the neotissue. While collagen fibers tended to align parallel to the print direction (i.e., at 0°C) irrespective of the bath stiffness (Figure 2B), the stiffer 2% support bath supported the greatest degree of fiber alignment (Figure 2A) and coherency (Figure 2D). We also investigated the size of the collagen fibrils through SEM imaging (Figure S6), where preferential alignment of the collagen fibers was observed on the surface of all groups. The diameter of the collagen fibril ranged between 20 and 60 nm (Figure 4C) after 4 weeks of in vitro culture, which is comparable to that observed in the cartilage of skeletally immature pigs [80].

### 2.5 Bioprinting of Anisotropic Musculoskeletal Tissues by Spatial Patterning Microtissues into Support Baths to Differing Stiffness

Musculoskeletal tissues, particularly those that endure mechanical loads in specific directions, exhibit preferential collagen fiber alignment to support their biomechanical functions. For example, tendons and ligaments have significantly higher tensile properties along the fiber direction [81]. In the meniscus, the predominance of circumferentially aligned type I collagen fibers provides the necessary stiffness (100–300 MPa) to enable this tissue to function within the knee joint [82]. Similarly, in AC, vertically oriented collagen fibrils in the deep zone protect the tissue against large compressive strains [83], while collagen fibers aligned parallel to the surface in the superficial zone play a crucial role in resisting high tensile stresses [84, 85]. To demonstrate the utility of this µT‐based bioprinting platform, we next sought to engineer larger tissue grafts with distinct phenotypes and anisotropic collagen fiber networks. As a proof of concept, we sought to replicate the distinct collagen arrangements observed in different musculoskeletal tissues, specifically the arcade‐like collagen alignment in AC [86], circumferentially aligned fibers in the menisci [87], and the linear collagen orientation found in ligaments [88]. To maintain anisotropy across multiple layers of printed µT filaments, we optimized filament spacing within the support bath to promote tissue compaction while preserving the desired collagen organization (Figure S7). For all these prints, the composition of the culture media remained constant (a chemically defined media supplemented with TGF‐β3), with only the stiffness of the bath changing for the bioprinting of the different tissue types.

**FIGURE 5:**
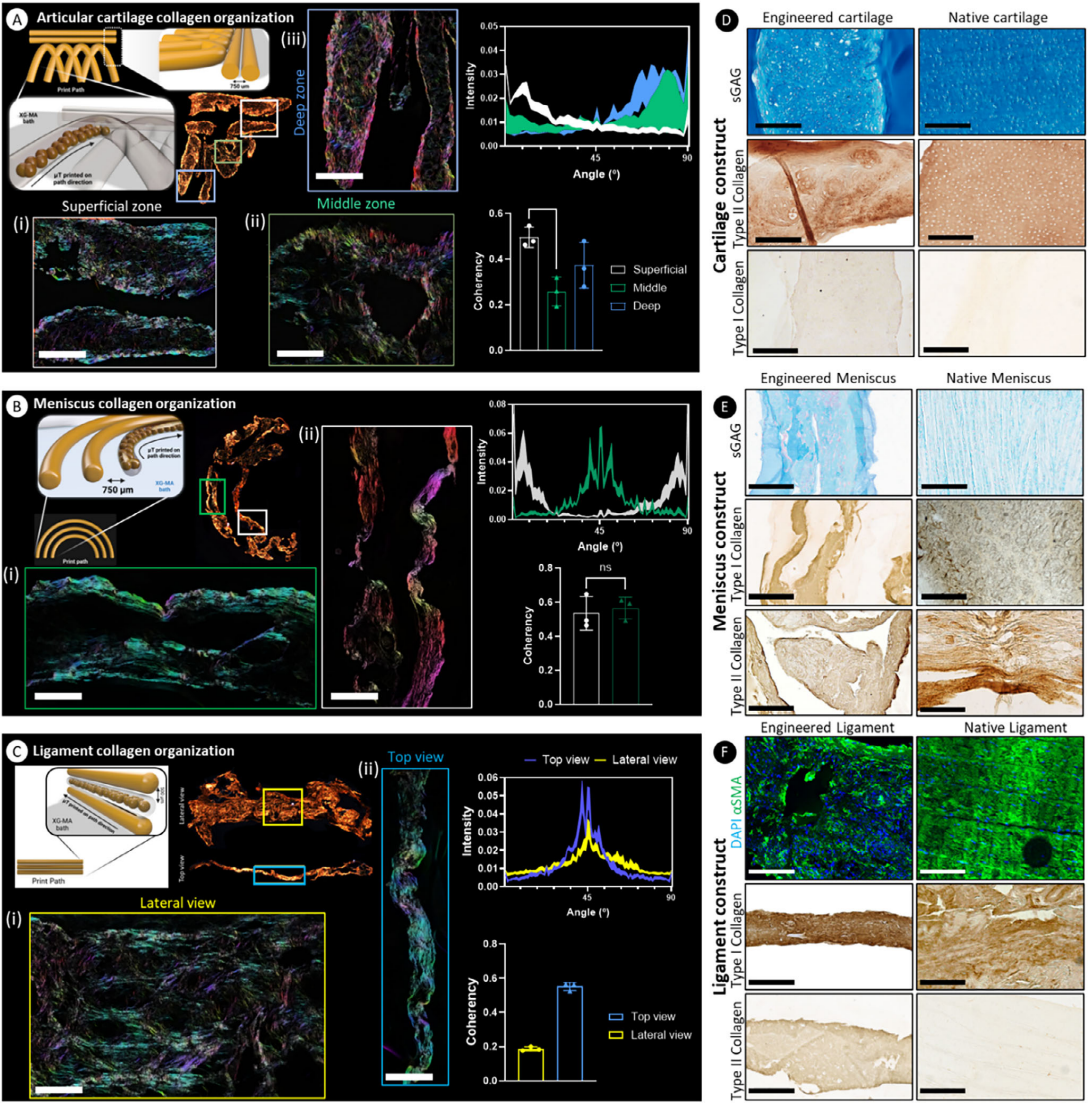
The bioprinting of scaled‐up anisotropic tissue grafts. A) Bioprinting of Benninghoff collagen arcades is typical of native AC. The collagen fiber orientation was quantified in the superficial (i), middle (ii), and deep (ii) regions of the tissue, indicating the development of highly anisotropic tissues. Coherency analysis values closer to 1 indicate that the fibers are aligned in one direction, while values close to 0 indicate the fibers are aligned in different directions. White indicates the color map of the superficial zone, green indicates the color map of the middle zone, and blue indicates the color map of the deep zone. Scale bar: 200 µm. B) Bioprinting of circumferential filaments to engineer a meniscal tissue. Images from two different regions (green (i) and white (ii) boxes) are provided, again demonstrating that the collagen fibers are aligning according to the printing path. Fiber coherency analysis indicates that most of the fibers are aligned in one direction. The green lines/bars represent the collagen fiber orientation within the regions highlighted within the green boxes (i). The white lines/bars represent the collagen fiber orientation within the regions highlighted within the white box (ii). Scale bar: 200 µm. C) Bioprinting of a 3‐layered anisotropic ligament tissue, where most of the fibers are aligned towards the 0°C direction in both the lateral (yellow color box (i)) and top (blue color box (ii)) view. Scale bar: 200 µm. A one‐way ANOVA test was performed, followed by Tukey's post hoc comparison to compare the means of each group at different time points. Significance was accepted when *p*<0.05. *Indicates *p*<0.05. ns: non‐significant. For all the graphs: *n* = 3. D) Histological and immunohistochemical assessment for the cartilage construct for sGAG, type II collagen, and type I collagen deposition compared to native AC. E) Histological and immuno‐histological assessment for the meniscal construct for sGAG, type I collagen, and type II collagen, compared to the native menisci. F) Histological and immunohistochemical assessment for the ligament construct for α‐SMA, type I collagen, and type II collagen compared to the native ligament. Scale bars: 200 µm for type I and II collagen and 100 µm for α‐SMA.

µTs were first extruded into a 0.5% XG‐MA support bath to generate an arcade‐like geometry characteristic of native AC (Figure S8A), where they fused and reorganized over time, aligning according to the print path, and generating a cartilaginous tissue rich in type II collagen and poor in type I collagen (Figure 5D). In the superficial zone of the resultant tissue, collagen fibers predominantly aligned parallel (0°C) to the surface of the construct, resembling that observed in native AC (Figure 5A(i). In the deep zone, fibers were primarily oriented at 90°C, reflecting the perpendicular alignment seen in native AC (Figure 5A(iii)). PLM quantification confirmed the superior tissue organization resulting from the support bath's temporal confinement, leading to effective µT fusion and ECM reorganization (Figure 5A). Furthermore, the soft XG‐MA support bath supported a chondrogenic phenotype, characterized by robust deposition of the hyaline cartilage specific matrix markers sGAGs and type II collagen (Figure 5D). For the meniscus construct, µTs were bioprinted in the intermediate stiffness 1% XG‐MA support baths in a circumferentially aligned pattern to replicate the organization seen in the meniscal ECM (Figure S8B). PLM analysis confirmed that the final construct displayed high collagen fiber anisotropy, with fiber orientation closely matching the printing direction (Figure 5B). The meniscus is composed of two distinct regions: the inner zone, which contains a mix of collagen types I and II, and the outer zone, primarily made up of collagen type I. Here, we successfully engineered a highly anisotropic meniscal tissue with uniform sGAG deposition and a high type I collagen content (Figure 5E)—characteristics typical of the outer zone, which accounts for over 80% of the meniscus body [89]. As a final proof of principle, we attempted to bioprint a ligament‐like tissue within the stiffer 2% XG‐MA support bath (Figure 5C,F) (Figure S8C). To this end, we bioprinted a three‐layered structure with parallel collagen alignment to replicate the ligament architecture (Figure 5C). Our results demonstrated that MSC‐derived µTs adapted to their surroundings, secreting type I collagen and expressing α‐SMA (Figure 5F), indicating (myo)fibroblastic differentiation [90]. Collectively, our results highlight that the physical boundaries provided by the support bath, applied to µT‐rich bioinks, can be used to direct neo‐tissue organization and enable the engineering of phenotypically distinct musculoskeletal tissues.

While stem cell‐derived microtissues and organoids have been bioprinted or used in bottom‐up modular assembly strategies, these approaches often face limitations in terms of shape fidelity and long‐term structural organization. For example, induced pluripotent stem cell (iPSCs)–derived organoids have been used as a ‘living bath’ into which vascular channels are bioprinted, achieving good construct stability and perfusion [95]. However, such systems typically lack internal architectural control, particularly with respect to collagen fiber alignment, which is critical to the biomechanical function of anisotropic musculoskeletal tissues. We previously addressed a related challenge by localising microtissues within 3D printed polycaprolactone scaffolds, enabling the development of scaled‐up cartilage that remodelled in response to the physical constraints provided by the supporting scaffold [96, 97]. Although this strategy supports the development of structurally organised tissue, a key limitation is the relatively slow degradation rates of the stiff polymeric scaffold, which can adversely affect tissue regeneration following implantation. Furthermore, synthetic biomaterials like polycaprolactone are known to promote fibrosis in vivo [98]. In the present work, we demonstrate the formation of anisotropic tissues within a mechanically tunable support bath, without the need for rigid polymer scaffolds. Our embedded bioprinting approach therefore, complements existing modular assembly techniques by combining control over tissue‐scale shape with internal guidance of collagen organization. A key limitation of this work is that the individual tissue filaments do not fully fuse in the support bath, which may negatively impact the overall functionality of the bioprinted construct. At the end of the culture period, the bioprinted tissue remains embedded in the photocrosslinked, non‐degradable XG‐MA support bath. Therefore, at present, these constructs are not yet suitable for direct implantation into damaged synovial joints, and further optimization is required to enable complete or controlled removal of the XG‐MA bath. Future work will explore the implementation of modified support baths that not only provides fusing µTs with sufficient mechanical support to direct their differentiation and remodeling, but which also degrade in a more programmable manner to ensure that residual bath material does not prevent the development of a cohesive, scaled‐up graft. In turn, this will also allow us to assess if the proposed bioprinting platform can generate biomechanically functional tissues suitable for in vivo implantation.

## 3 Conclusion

In summary, this novel bioprinting platform, which leverages a mechanically tunable support bath, creates a dynamic environment that enables the precise spatial patterning of µT‐laden bioinks. We demonstrate that the physical environment created by the surrounding support bath can modulate the phenotype of the printed µTs, enabling the engineering of different musculoskeletal tissues by simply varying the support bath stiffness. Remarkably, this approach not only supports distinct musculoskeletal phenotypes but also controls µT (re)modelling and subsequent ECM organization, thereby enabling the engineering of anisotropic grafts with user‐defined collagen architectures. Furthermore, we demonstrate the versatility of this platform by bioprinting a range of different musculoskeletal tissues, including articular cartilage, meniscus, and ligament grafts. These findings support the continued development of bioprinting strategies that more closely mimic normal developmental processes to better engineer functional tissues or organs. In the future, such bioprinting platforms could potentially provide implantable, functional grafts at scale for a range of different regenerative medicine applications.

## 4 Experimental Section

### 4.1 Modification of Xanthan Gum with Methacrylic Group (^1^H‐NMR) and Support Bath Preparation

Xanthan gum (XG) (Sigma) was dissolved at 0.5% (w/v) into ultra‐pure water and stirred until completely dissolved. Once dissolved, glycidyl methacrylate (40 mL) was added to the XG solution and stirred overnight at 60°C, protected from light. The solution was now dialyzed for 7 days against DI with water changes twice a day, freeze‐dried for 48 h, and stored at −20°C for long‐term storage. To ensure sterilization, aliquots of XG‐MA were weighted and placed into a 50 mL tube and sterilized in an ethylene oxide gas sterilizer (Andersen Products) for 12 h. 2 days prior printing, the material was dissolved into phenol free DMEM (Gibco) supplemented with penicillin (100 U mL^−1^), streptomycin (100 µg mL^−1^) (P/S) (both Gibco) and let it rotate at 40 rpm at RT for 2 days. The day of printing, Lithium phenyl‐2,4,6‐trimethylbenzoylphosphinate (LAP) (Sigma), was added for a final concentration of 0.25% (w/v) into the XG‐MA solution and vortexed. To ensure the removal of the bubbles, the bath was centrifuged at 2500 x *g* for 5 min at RT, prior to printing. For each print, XG‐MA (4 mL) was pipetted into a 6‐well plate with a pipette for viscous liquids (Microman E, Gilson) to ensure no formation of bubbles during the pipetting.

### 4.2 Rheological Assessment of Bioink and Support Baths

Rheological characterizations were conducted on an MCR 102 Rheometer (Anton‐Paar, Hertford Herts, UK) equipped with a Peltier element for temperature control. A plate‐plate geometry with a diameter of 25 ​mm (PP25) was used in all the tests as previously described [94]. The viscosity as a function of shear rate (0.1–1000 s−1) was measured at a constant temperature of 4 ​°C for the gelatin (Gelatin Type B) (Sigma) bioink, and at RT for the XG‐MA support bath. To investigate the linear viscoelastic region (LVR) and determine the yield stress, an amplitude sweep test was carried out at RT. An angular frequency between 1 and 100 rad s^−1^ was applied to the sample to determine G` and G`` curves. The G` and G`` crossover point was taken as the yield point where the material behavior transitions from solid to liquid‐like behavior [91]. To evaluate the shear strain range (1%–100%) and determine the solid and liquid‐like state of the different inks, we performed a dynamic sweep stress test. Five intervals at a constant shear rate were performed to determine the thixotropy behavior of the support bath and investigate the time dependency of the support bath. Here, the viscosity was measured in transient shear rate step tests at RT. First, samples were sheared at a shear rate of 0.03 s^−1^ for 30 s, then the shear rate increased to 10 s^−1^ for 30 s, and again, the shear rate decreased to 0.03 s^−1^ for 30 s. Bioinks and support baths were maintained under high‐humidity conditions to minimize dehydration during testing, and all rheological measurements were carried out in triplicate.

### 4.3 Compression testing of XG‐MA hydrogels

Unconfined compression tests were performed on cylindrical samples prepared using a 5 mm PDMS mold and crosslinked under UV light for 4 min. After demolding, samples were immersed in PBS to prevent dehydration and subsequently compressed at a rate of 5 mm·s^−^^1^ using a twin‐column Zwick universal testing machine (Zwick Roell) equipped with a 10 N load cell. A preload of 0.01 N was applied to the hydrogels prior to testing. Compression was continued until sample failure, and the Young's modulus was calculated as the slope of the linear region of the stress–strain curve up to 70% strain.

### 4.4 Isolation and expansion of caprine BM‐MSCs

Caprine bone marrow mesenchymal stem cells (MSCs) were isolated from the sternum of skeletally mature female goats as previously described [11]. Briefly, bone marrow was cut into smaller fragments and vortexed in high‐glucose DMEM (hgDMEM, Gibco) supplemented with penicillin/streptomycin and 10% fetal bovine serum (FBS, Gibco), hereafter referred to as expansion medium (XPAN), to release cellular components. At ∼80% confluency, cells were cryopreserved in liquid nitrogen for long‐term storage. For further expansion, MSCs were reseeded at a density of 5 × 10^3^ cells·cm^−^^2^ under 5% O_2_ and expanded up to passage 3 (P3) for all bioprinting experiments.

### 4.5 Fabrication of MSC Microtissues

Microtissues (µTs) were fabricated as previously described [92]. Briefly, 3D‐printed mold stamps containing 1,889 micro‐resections and 4% (w/v) ultrapure agarose (Sigma) were sterilized in an autoclave at 120°C for 20 min. The agarose was then poured into each well of a 6‐well plate, molded with the stamps, and allowed to cool for 15 min. The resulting agarose microwell arrays were soaked overnight in XPAN at 37°C under physioxic conditions (5% O_2_, humidified atmosphere). MSCs were seeded into the microwells at a density of 7.556 × 10^5^ cells·mL^−^^1^ (5 mL per well), yielding 1,889 µTs with approximately 2,000 cells per µT, and centrifuged at 700 × g to collect cells at the bottom of each well. The µTs were then cultured in chondrogenic medium (CDM) consisting of hgDMEM GlutaMAX supplemented with penicillin/streptomycin, sodium pyruvate (100 µg·mL^−^^1^), L‐proline (40 µg·mL^−^^1^), L‐ascorbic acid‐2‐phosphate (50 µg·mL^−^^1^), linoleic acid (4.7 µg·mL^−^^1^), bovine serum albumin (1.5 mg·mL^−^^1^), 1× insulin−transferrin−selenium (ITS), dexamethasone (100 nM) (all Sigma), and human transforming growth factor‐β3 (TGF‐β3, 10 ng·mL^−^^1^; Peprotech, UK) [11]. After 2 days of culture, µTs were harvested for bioprinting. Media was gently flushed over the agarose surface with a micropipette and collected into a 50 mL tube, as previously described [11]. The density of the µT suspension was quantified, and the required volume was adjusted to achieve the desired concentration for each bioprinting experiment.

### 4.6 Bioink Preparation and Bioprinting Parameters

Sterile gelatin type B (1% w/v) was dissolved in hgDMEM for 30 min at 37°C in a water bath the day prior to printing. After 30 min, gelatin is removed from the water bath and placed at 4°C overnight for gelation. µTs are harvested from the agarose microwells according to methods described before [11], placed in a 50 mL tube with media, the tube is centrifuged at 650 x *g* for 5 min at RT to collect all the µTs at the bottom of the tube. Gelatin was then added to reach desired µTs density for bioprinting. Afterwards, the bioink is carefully loaded into the cartridge and placed into the fridge for 15 min prior bioprinting, in sterile conditions. The bioprinting is carried out using a syringe pump printhead (Cellink, Sweden), with extrusion rate of 6 uL s^−1^ and print speed of 2 mm s^−1^. Post‐printing, the XG‐MA support bath was UV‐cured under sterile conditions using a 395 nm lamp (10 mW cm^−^^2^) for 4 min.

### 4.7 Live/Dead assay

Cell viability was assessed using a live/dead assay kit (Invitrogen) as previously described [11]. Briefly, constructs were rinsed with PBS, incubated for 1 h at 37°C in a staining solution containing calcein (2 µM, excitation 485 nm, emission 530 nm) and Ethidium Homodimer (4 µM, excitation 530 nm, emission 645 nm), and then rinsed again in PBS to remove excess dye. Samples were imaged with a Leica SP8 confocal microscope, and maximum projection z‐stacks were generated to evaluate viability throughout the tissue depth.

### 4.8 Scanning Electron Microscopy (SEM) imaging and analysis

Samples were fixed in 0.1 M cacodylate buffer (Sigma) at 4°C for a minimum of 12 h. The samples were enzymatically treated according to methods described before [80] to remove any excess of GAGs that could disrupt the imaging of the collagen fibers. They were then washed with 1X PBS and dehydrated in graded ethanol baths series (50%–100% EtOH) and dried using critical point drying method to better preserve the surface structure of the biological sample. Samples were imaged using a Zeiss Ultra Plus.

### 4.9 Histological Evaluation

Briefly, samples were fixed in 4% paraformaldehyde (PFA) overnight at 4°C. After fixation, they were dehydrated through a graded ethanol series (50%–100% v/v), cleared in xylene, and embedded in paraffin wax (Sigma) as previously described [11]. Sections of 5 µm thickness were cut, rehydrated, and subjected to histological staining. Haematoxylin and eosin (H&E; Sigma) was used to assess tissue morphology, ECM, and nuclei. Sulfated glycosaminoglycan (sGAG) content was stained with 1% (w/v) Alcian Blue (AB) 8GX in 0.1 M HCl and counterstained with 0.1% (w/v) nuclear fast red for cell distribution. Collagen deposition was visualized using 0.1% (w/v) Picrosirius Red (PR; Sigma). Stained sections were mounted in Pertex (Avantor) and imaged with an Aperio ScanScope slide scanner (Leica, Germany).

### 4.10 Immunohistochemical Staining and Quantification

Immunohistochemistry was performed for collagen type I (Abcam ab90395, 1:400) and collagen type II (Santa Cruz sc52658, 1:400) as previously described [11], as well as for α‐SMA (Abcam ab124964, 1:200) and YAP (Abcam ab3472, 1:200). Wax‐embedded sections were rehydrated, washed with PBS, and, for α‐SMA and YAP, subjected to antigen retrieval in sodium citrate buffer (10 mM, 0.05% Tween‐20, pH 6) at 80°C for 20 min. Samples were blocked with 10% donkey serum in PBS + 0.05% Tween‐20, incubated overnight at 4°C with primary antibodies, washed, and then incubated with secondary antibodies (1:200) for 1 h at room temperature in the dark. Sections were mounted with Fluoroshield containing DAPI (Sigma), sealed with nail polish, and stored at 4°C until imaging. Fluorescence intensity was quantified from three ROIs per sample (*n* = 3) using ImageJ and normalized to the number of nuclei.

### 4.11 RNA isolation and quantitative Real‐Time PCR

At 7 and 28 days after µT bioprinting, samples were washed three times with PBS and snap‐frozen for further processing. RNA was extracted using the Trizol (Sigma) method according to the manufacturer's instructions [11]. After chloroform extraction, RNA was resuspended in RNase‐free water (Gibco) and stored at −80°C. cDNA was synthesized from 500 ng of RNA using the High‐Capacity RNA‐to‐cDNA Kit (Applied Biosystems) in a 20 µL reaction (37°C for 1 h, 95°C for 5 min, 4°C for 5 min). Real‐time PCR was performed on an Applied Biosystems instrument using TaqMan Fast Advanced Master Mix (ThermoFisher). Goat‐specific TaqMan probes (ThermoFisher) were used for target gene amplification, and 18S rRNA served as the housekeeping gene. The qPCR cycling program was: 50°C for 2 min, 95°C for 10 min, followed by 40 cycles at 60°C for 1 min. Gene expression was quantified using the 2^−ΔΔCt^ method, normalized to 18S rRNA levels, and compared with MSC monolayer controls.

### 4.12 Filament Diameter Measurements and Polarized Light Microscopy Analysis

The diameter of bioprinted µTs was measured using an inverted microscope (Primo Vert, Zeiss, USA) with a 4× objective. Images were acquired at defined time points up to 28 days post‐printing, and diameters were quantified with ImageJ software (Wayne Rasband and contributors, USA). For each replicate, four independent measurements were obtained per construct. For polarized light microscopy (PLM), paraffin‐embedded tissue sections were stained with 0.1% (w/v) Picrosirius Red (Sigma–Aldrich), mounted with Pertex (Sigma–Aldrich), and imaged using an Olympus BX41 polarizing microscope equipped with a MicroPublisher 6 CCD camera and a U‐CMAD3 adaptor. Collagen fibril orientation, dispersion, and coherency were analyzed using the OrientationJ and Directionality plugins in ImageJ.

### 4.13 Statistical Analysis and Graphical Results

Statistical analyses were performed using the software package GraphPad Prism (version 10.4.1). All data were pre‐processed before analysis. Outliers were identified and excluded using the ROUT method where applicable. Statistical tests used to assess the of data or to compare groups are indicated in figure legends. For experiments involving comparison of more than two groups under a single experimental factor, one‐way ANOVA followed by Tukey's post hoc test was applied. For experiments involving two variables, two‐way ANOVA followed by Tukey`s post hoc test was used to evaluate interaction effects and main effects. When groups were compared, significance was accepted at a level of *p* < 0.05. Statistical significance is indicated as follows: *p<0.05, **p<0.01, ***p<0.001, **** *p*<0.0001. Results are expressed as mean ± standard deviation. The sample size (n) for each experiment is reported in the corresponding figure legends were produced with GraphPad Prism.

## Conflicts of Interest

The authors declare no conflicts of interest.

## Supporting information




**Supporting File**: advs73969‐sup‐0001‐SuppMat.docx.

## Data Availability

The data that support the findings of this study are openly available in Biorxiv at https://doi.org/10.1101/2025.05.01.651636, reference number 651636.
