# Supplementary material for: Bioprinting of Microtissues Within Mechanically Tunable Support Baths to Engineer Anisotropic Musculoskeletal Tissues
Source: Adv Sci (Weinh). 2026 Feb 6;13(18):e09313. doi: 10.1002/advs.202509313 (PMC13042905; doi:10.1002/advs.202509313)
Supplement: Supplementary file 1 — Supporting File: advs73969‐sup‐0001‐SuppMat.docx. [file ADVS-13-e09313-s001.docx]

**4D Bioprinting of Microtissues Within Temporally Adapting Support Baths to Engineer Anisotropic Musculoskeletal Tissues**

Francesca D. Spagnuolo^1,2^, Gabriela S. Kronemberger^1,2^, Daniel J. Kelly^1,2,3,4*^

^1^Trinity Centre for Biomedical Engineering, Trinity Biomedical Sciences Institute, Trinity College Dublin, Dublin, Ireland.

^2^Department of Mechanical, Manufacturing and Biomedical Engineering, School of Engineering, Trinity College Dublin, Dublin, Ireland.

^3^Department of Anatomy and Regenerative Medicine, Royal College of Surgeons in Ireland, Dublin, Ireland.

^4^Advanced Materials and Bioengineering Research Centre (AMBER), Royal College of Surgeons in Ireland and Trinity College Dublin, Dublin, Ireland.

*Corresponding author

**Keywords:** microtissues, bioprinting, support bath, anisotropy, stiffness

**Supporting Information**


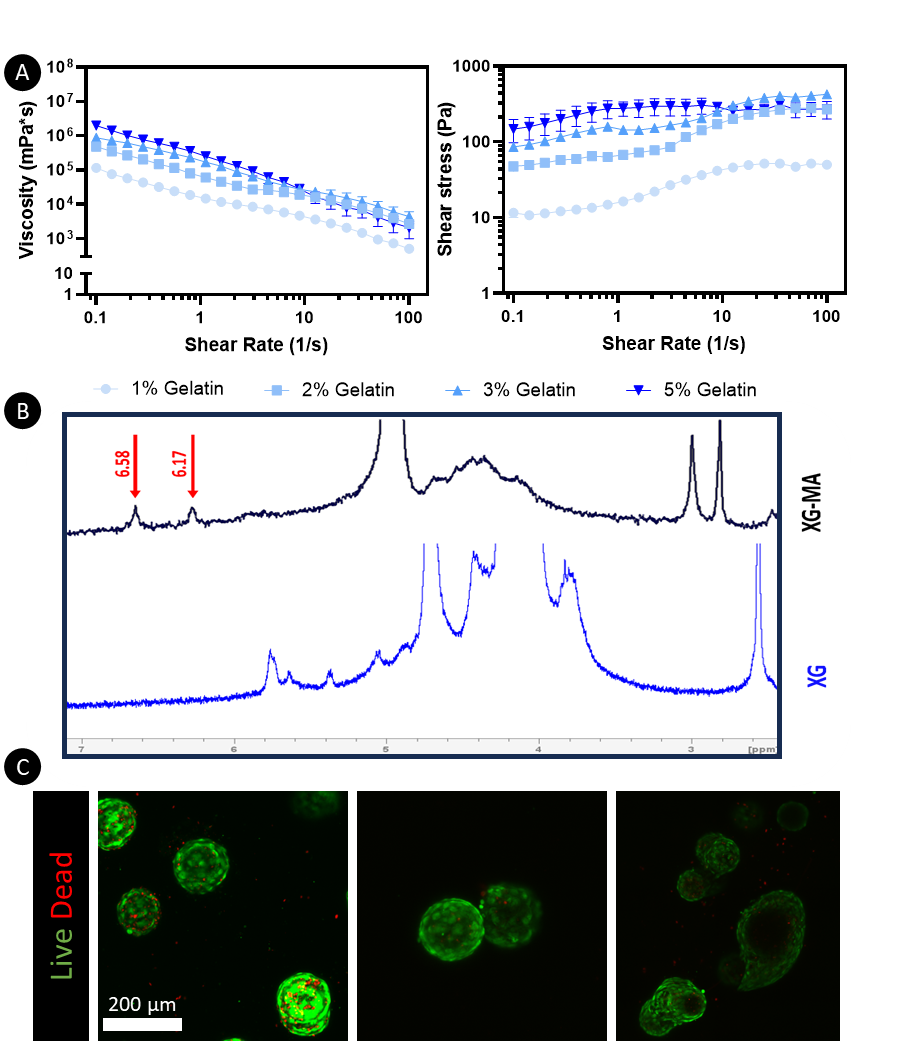


**Figure S1**. **Characterization of the XG-MA modification, gelatin viscoelastic behavior and cell viability, crucial for the successful development of the 4D bioprinting platform.** A) Viscoelastic behavior of gelatin bioink at different concentrations (5%, 3%, 2% and 1%) showing shear thinning behavior critical for bioink development. B) Proton nuclear magnetic resonance spectra (^1^H-NMR) confirming the successful modification of XG with a methacrylic group (-C=C). Red arrows indicate the peaks of the methacrylic group at 6.58 and 6.17 ppm. C) Live/dead of microtissues (2000 cells/µT) encapsulated in gelatin 1% prior printing show high viability of microtissues. Green: Calcein (Live cells), Red: Ethidium Bromide (dead cells). Sb: 200 µm.


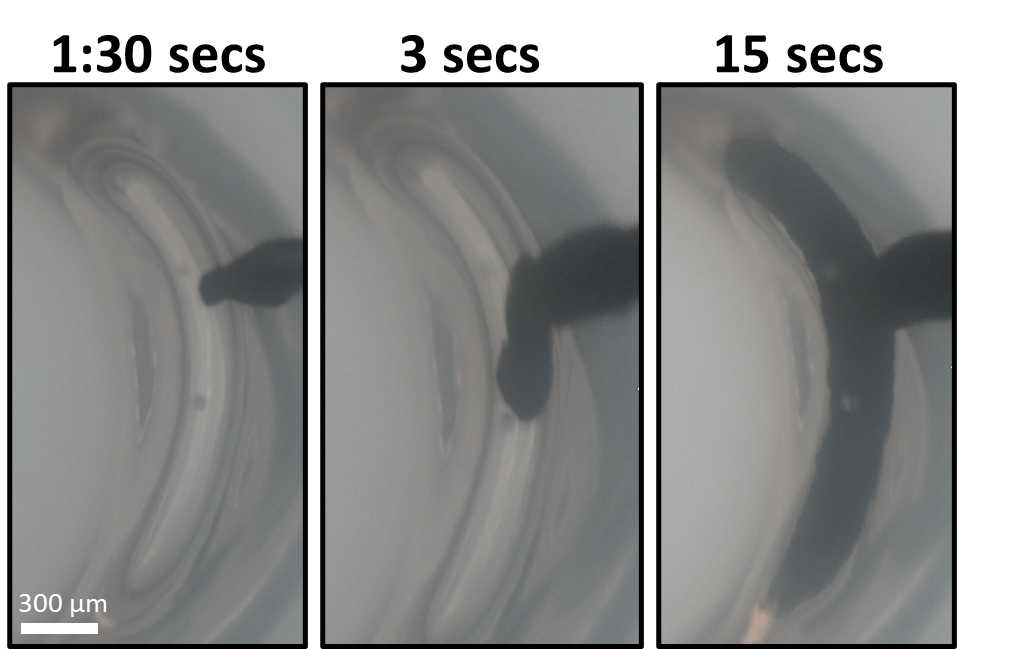


**Figure S2. Perfusion of black dye through a void channel formed after the diffusion of a printed 1% gelatin filament within the microenvironment.** Following printing and UV curing of the support bath, the gelatin bioink liquefied and diffused into the surrounding microenvironment, leaving behind a void channel. This channel was subsequently perfused with black dye to confirm that the channel remained open, illustrating how gelatin diffusion supports the temporal reduction in filament diameter over time. Scale bar: 300 µm.

**
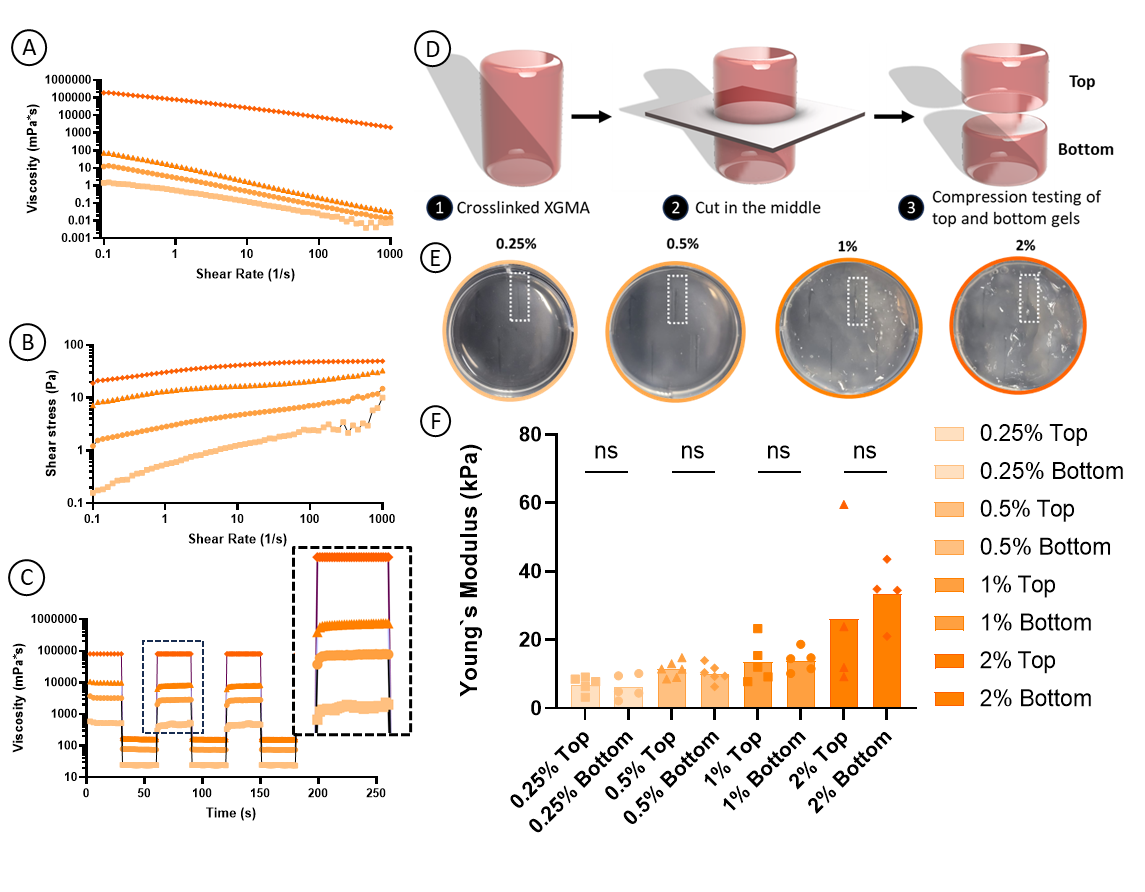
**

**Figure S3. Rheological properties of XG-MA at different concentrations and UV-curing depth influence on the bath stiffness.** A) Viscosity dependency on shear rate of the support bath at different concentrations, from low shear rate (0.1 s^-1^) to very high shear stress (1000 s^-1^) demonstrating shear-thinning properties B) Shear stress dependency on shear rate of the support bath at different concentrations, from low (0.1 s^-1^) to high (1000 s^-1^) shear stress C) Stress-relaxation behavior of the XG-MA support bath at different concentrations to assess the recovery of the material post applying high shear stress D) Graphical representation of mechanical testing of both halves (top and bottom) of the support bath post UV exposure to assess UV curing depth E) macroscopic pictures of acellular (1% gelatin only) printed filaments into support baths at different concentrations, with different optical properties F) mechanical testing of both halves (top and bottom) of each gel to assess curing depth dependency of all concentrations of the support bath post-UV exposure (4 min). A two-way ANOVA test was performed followed by Tukey’s post comparison to compare the means of each group at different time points. Significance was accepted when p<0.05. ns: non-significant.

**
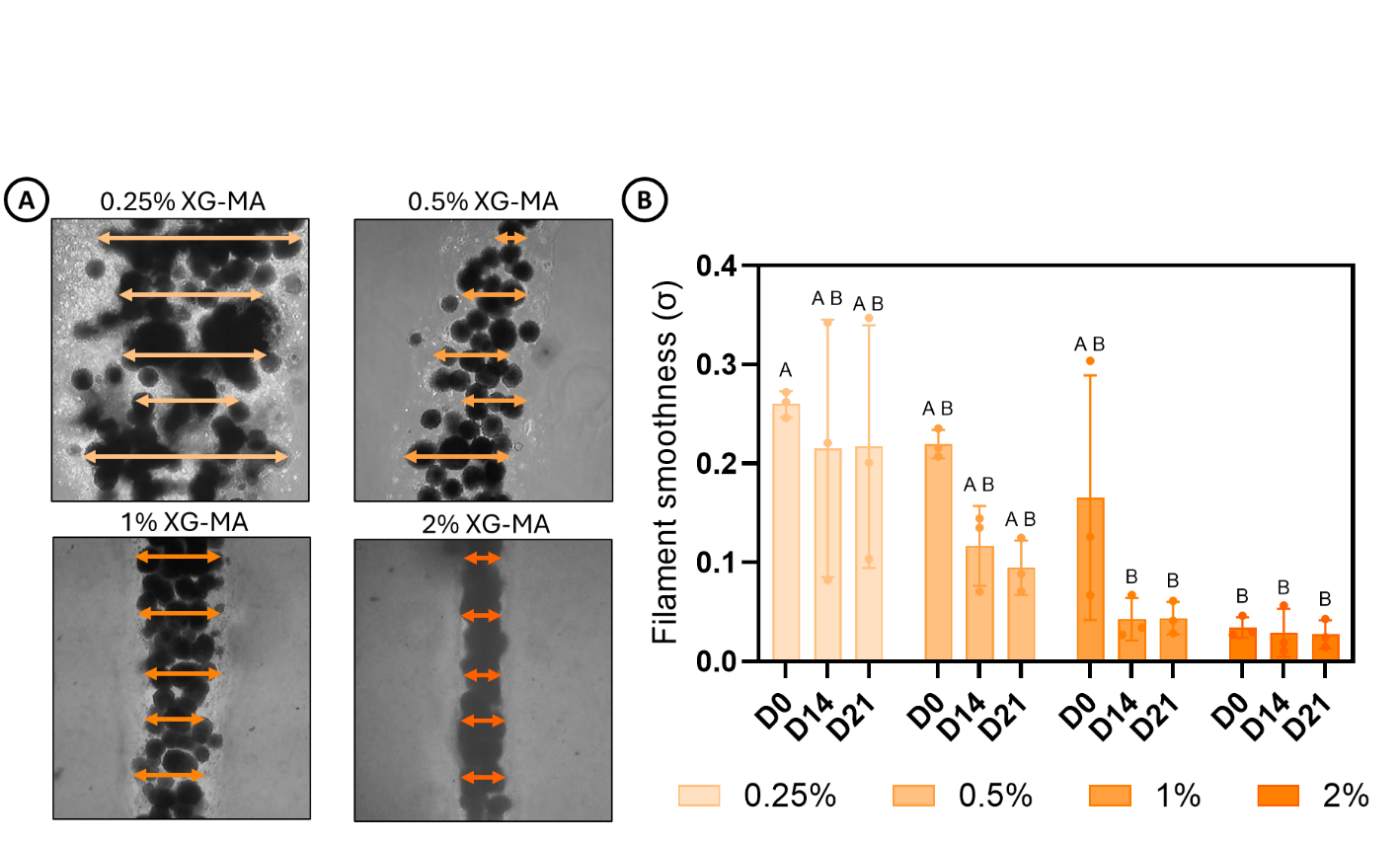
**

**Figure S4. Quantification of filament smoothness**. A) Brightfield microscopy images were used to assess local diameter variations of printed microtissue filaments. For each filament, five diameter measurements were taken at distinct regions along its length (n = 3 images per condition). The brightfield images represent the printed microtissues at day 0 under different conditions (refer to Figure 1). B) The standard deviation of these measurements was calculated as an index of smoothness, with lower values indicating more uniform and smoother filaments, and higher values reflecting increased irregularity. This parameter helps assess microtissue fusion and filament compaction post-printing. 2-way ANOVA followed by Tukey`s post hoc comparison test was performed to assess the differences between the groups; significance was accepted when p<0.05. Statistically comparable groups are labeled using compact letter display where groups sharing the same letter are not significantly different (p>0.05).

**
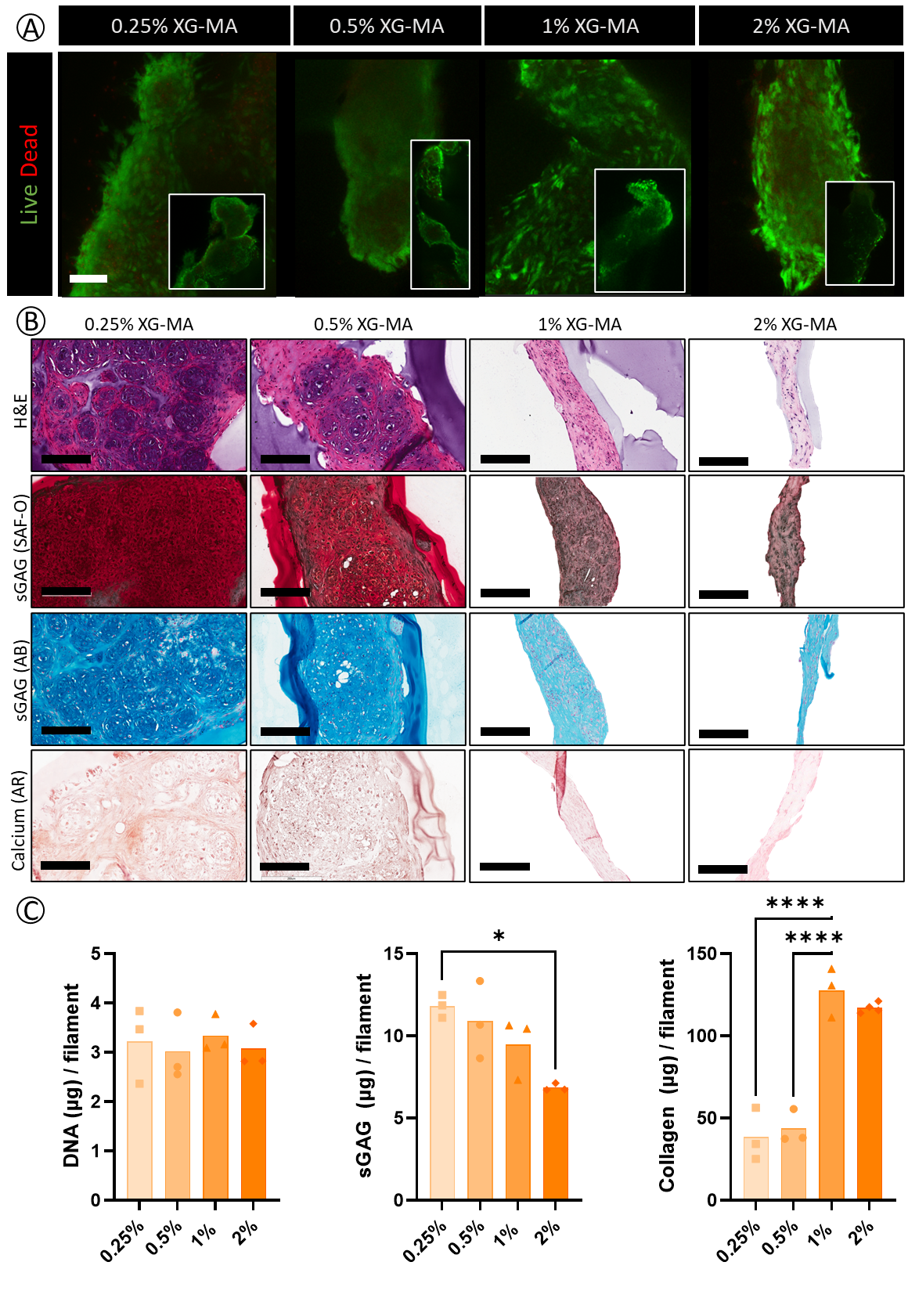
**

**Figure S5. Stiffness of the supporting bath post printing influences the deposition of sGAGs and matrix re(modelling) over time in culture.** A) Live/dead staining of printed microtissues into XG-MA support bath, 7 days after printing. Green: calcein AM; Red: Ethidium Bromide. Sb: 100µm. B) Histological evaluation of the printed microtissues after 28 days in culture with H&E, Safranin-O (SAF-O) and Alcian Blue to evaluate sGAGs deposition, which decreases with the increase in stiffness of the XG-MA support bath. Sb: 200 µm. C) Biochemical quantification of total DNA, sGAG and collagen per printed filament. Statistical difference is determined using a two-way ANOVA test with Tukey’s post comparison test. Significance was accepted with p<0.05. * indicates p<0.05, **** indicates p<0.0001.


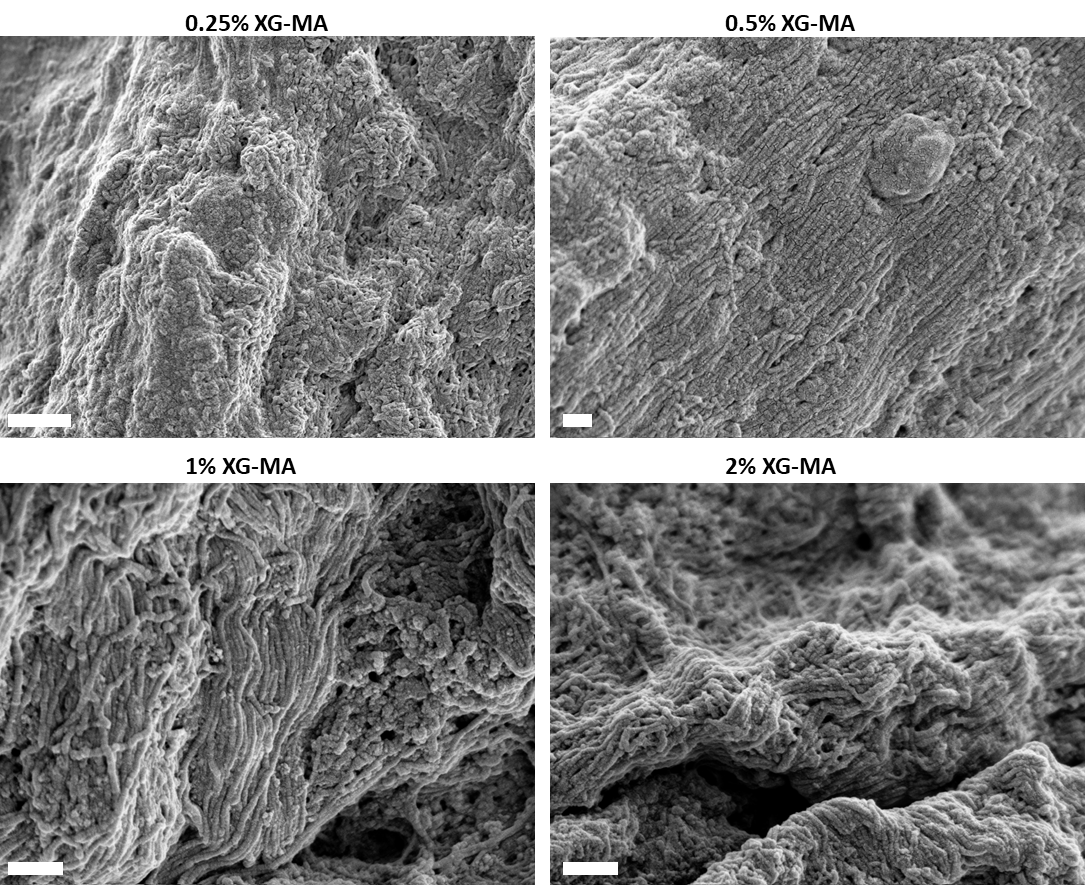


**Figure S6. (Re)modelling of the collagen fibers along the printing direction after 28 days in culture.** Scanning electron microscope (SEM) pictures of bioprinted microtissues to show the ECM organization and (re)modelling, driving tissue maturation and differentiation. Sb: 10 µm.


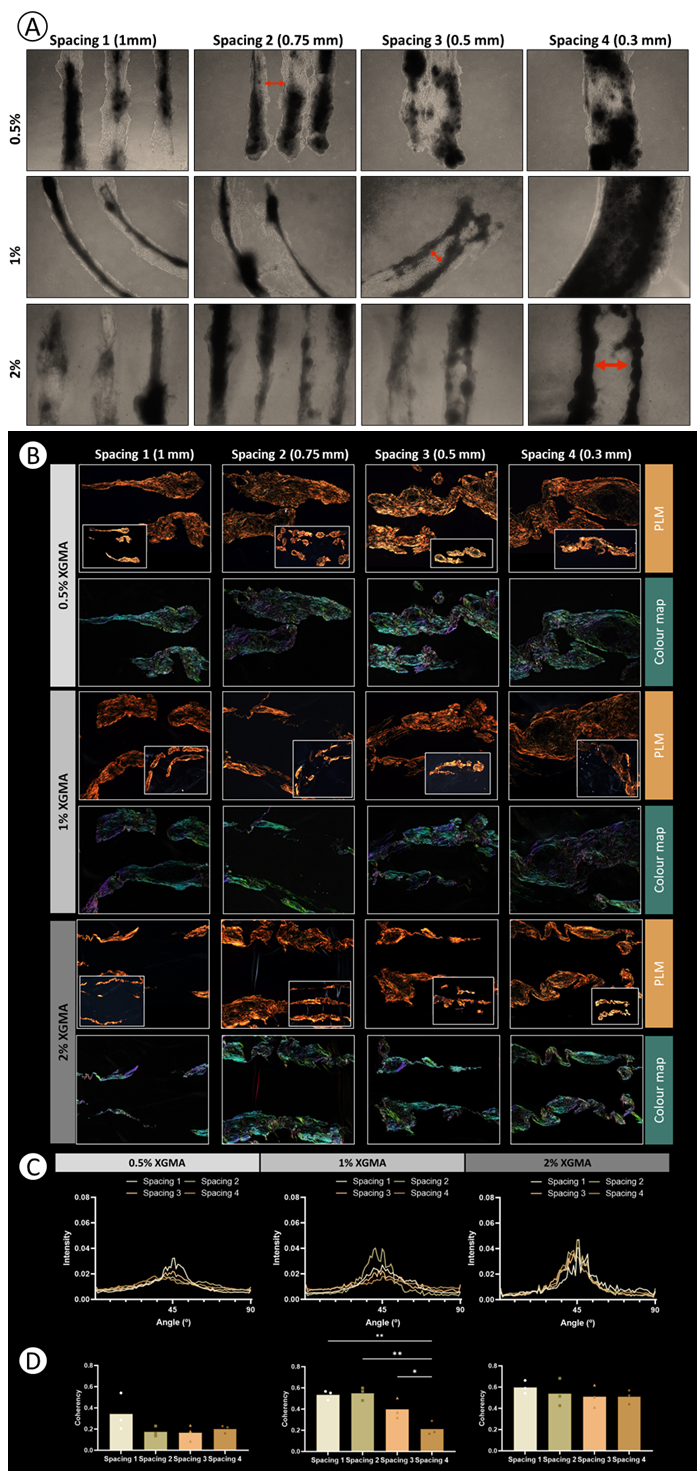


**Figure S7. The spacing (distance) between filaments post printing modulates the anisotropy of a scaled up bioprinted tissue construct**. A) Brightfield microscope pictures of bioprinted microtissues 28 days post printing showing fusion of the microtissues and adequate distance between each filament. B) Polarized Light Microscope images of bioprinted filaments at different distancing after 28 days of culture in TGF-β3 supplemented media, in support bath with variable stiffness. C) Mean average of the collagen fibers indicates that the spacing of the filaments into a scaled-up constructs influences the final anisotropy of the printed construct. D) Fiber coherency shows highly aligned tissues. Color maps are generated from PLM images. Here, color hue is used to indicate fiber orientation where blue/cyan indicated fibers oriented at 0 degrees and pink/red indicated fibers oriented at 90 degrees. For all the graphs a one-way ANOVA test was performed followed by Tukey’s post comparison to compare the means of each group at different time points. Significance was accepted when p<0.05. * Indicates p<0.05. **Indicates p<0.01. For all the graphs: n=3.

**
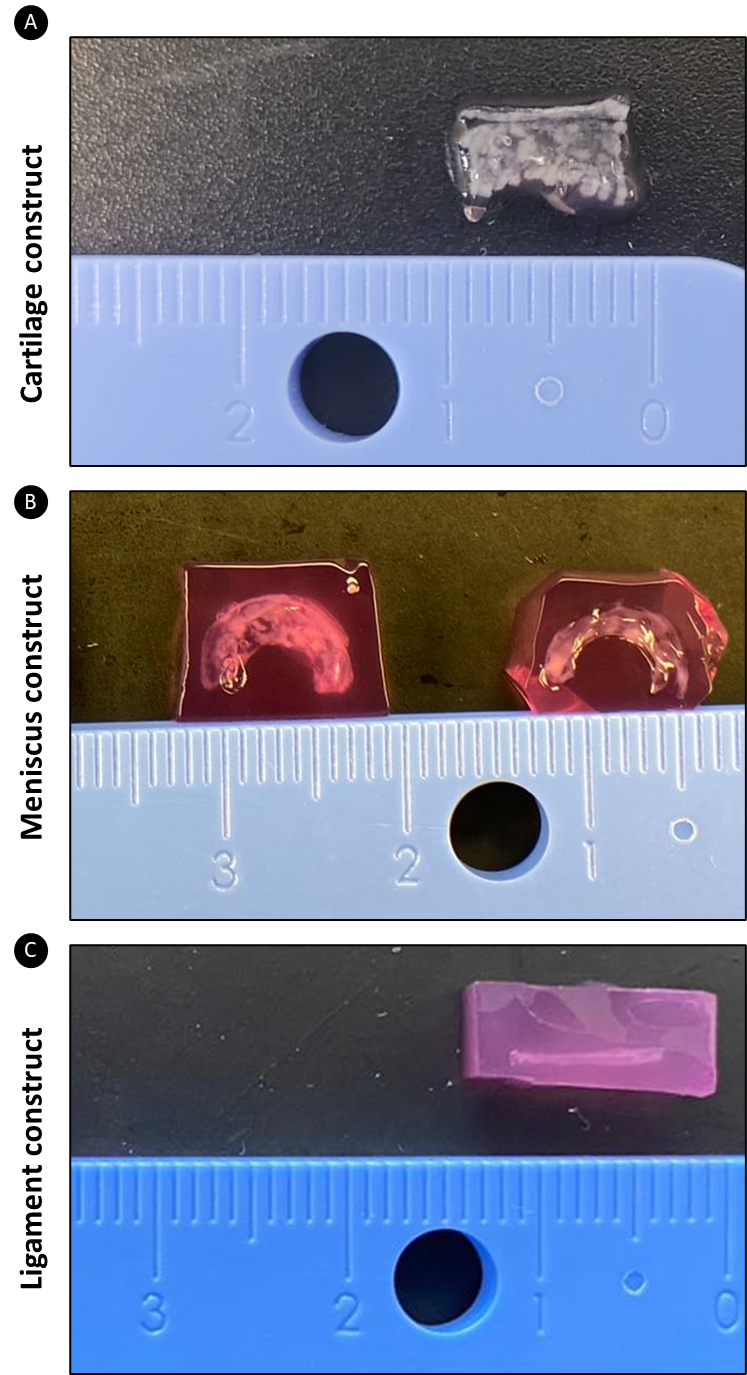
**

**Figure S8**. **Macroscopic images of scaled up tissue grafts**. A) Cartilage construct B) Meniscus constructs C) Ligament tissue construct.
